# Antiseptic Midwifery

**Published:** 1889-11

**Authors:** F. W. McRae

**Affiliations:** Atlanta, Ga.


### ANTISEPTIC MIDWIFERY.

F. W. McRAE, M. D., ATLANTA, GA.

In this day of antiseptic surgery and the general prevalence of antiseptic idea, I shall not discuss as *sub judice* the necessity of using antiseptics in obstetric practice.

The results obtained in all the large hospitals of this country and Europe, since the introduction of antiseptics into the lying-in wards, are sufficiently conclusive to convince the most skeptical observer. Before antiseptics were used, in October '83, by Garrigues, at the "New York Maternity Hospital, the average mortality for the preceding nine years was 4.17 per cent.; during the last six months 8 per cent.; during the last month it was 20 per cent., and in nearly 16 per cent. the cause of death was sepsis."

During the years 1884 to 1888 inclusive, there were 2,371 deliveries in above institution, with an average mortality of 1.01 per cent. from all causes, and of 0.25 per cent. from sepsis.

In discussing this subject I shall not confine myself simply to chemical disinfectants, but shall also consider briefly those equally important details of "antiseptic cleanliness," as applicable to private practice. My individual experience has been so gratifying to myself and to my patients as to compensate me many times over for the extra care and attention required.

I append reports of a few cases as illustrative of the results obtained by the use of antiseptics in my practice:

CASE I.—Mrs. J., *æt.* 35, black, multipera, previous health good; during eighth month of gestation was seized with violent convulsions in the night of the — — —. The convulsions followed each other in quick succession, until the attending physician was called in, and succeeded in controlling them temporarily by the administration of chloroform and chloral, and left patient for the night.

He was called hurriedly the next morning and found the convulsions had returned with increased frequency and violence, the woman being in a state of coma. I was then called in, and we decided to deliver immediately. Upon examination the os was found undilated but dilatable, and after considerable effort we succeeded in enlarging it sufficiently to admit the forceps, which I applied and slowly and carefully completed the delivery of a living child. Hands and instruments were of course thoroughly disinfected. The after treatment was conducted upon strict antiseptic principles, and the recovery was all that could have been desired. The temperature at no time was higher than 99° Fahrenheit.

CASE II.—Black, *æt.* 18, primipera, previous health good; had been in labor 18 hours when I was called in by attending physician, on account of uterine inertia, which had lasted several hours. Upon examination I found the os completely dilated and the head resting upon the floor of the pelvis. After anæsthetizing the patient, I applied the forceps and completed the delivery of a very large living child without accident. The subsequent treatment was with antiseptic precautions, but the attending physician having a suppurating onychia, infected the patient, and she had considerable fever for two or three days, otherwise making an excellent recovery.

CASE III.—Mulatto, *æt.* 25. Primipera, previous health good; labor progressed nicely until just before the dilation was completed, when the membranes ruptured, the pains increasing in force and frequency, wedged the anterior lip of the os firmly between the occiput and the pubes, causing it to become congested and thickened to such an extent as to prevent further descent of the head. After waiting for quite awhile, attempting in the meanwhile to press upon the obstructing lip, I sent for two medical students to assist me, anæsthetized the patient, applied the forceps and completed a very tedious delivery without accident, other than a very slight tearing of the fourchette.

The patient made an uninterrupted recovery without the slightest elevation of temperature or other unpleasant symptoms.

CASE IV.—White, *æt.* 35, multipera, very fleshy, weighing 240 pounds; general health good, abortion at the end of the fourth month from over-exertion. When called in I found the fœtus in the vagina, but the placenta firmly adherent, and removed with very great difficulty by the introduction of two fingers into the womb. After curetting the womb thoroughly, I washed it out with a 1:5000 bichloride solution, washed off the external parts with a 1:1000 solution, applied the antiseptic pad and removed all soiled clothing and bedding.

Recovery without fever or other complications.

The cases reported above simply show the results which may be obtained even under the most unfavorable surroundings in private practice. And while none of the patients might have died under the old plan of treatment, without any attempt at absolute cleanliness or the use of antiseptics, they would certainly have suffered many discomforts which were avoided by the use of the latter.

Assuming then that antiseptics should be used, the question narrows itself to which are best and safest.

Garrigues, in a paper published in the *American Journal of the Medical Sciences*, August, 1889, on "Corrosive Sublimate and Creolin in Obstetric Practice," has discussed the subject very thoroughly. He has collected from all sources twenty-two deaths from the use of bichloride of mercury during the lying-in period. In most of the cases reported, excessive quantities or too strong solutions were used. He concludes from a careful analysis of the cases, that the danger is greater after abortion than after labor at term. He condemns the use of intra-uterine injections of greater strength than one to five thousand, of which not more than a quart to a quart and a half should be used at a time under ordinary circumstances. Of creolin he speaks very favorably, and sets forth clearly the advantages which it possesses over both carbolic acid and corrosive sublimate. "In several respects creolin recommends itself particularly to the obstetrician. Its property of making surfaces slippery is of great value in operations, especially turning. Its great hæmostatic power makes it a most desirable drug for intra-uterine injections immediately after delivery."

Different experimenters have obtained somewhat different results as to its comparative antiseptic value, some placing it above and others below carbolic acid in the list of antiseptics. These different results are perhaps due to a lack of uniformity in the preparations employed.

Creolin is a dark syrupy liquid which makes an opaque emulsion with water, and is claimed to be entirely innocuous in the proportions in which it is ordinarily used as a disinfectant.

A two per cent. solution is strong enough for intra-uterine injections.

In the use of antiseptics in private practice among the better classes, some minor details, as carried out in hospitals, are not essential; especially is this so, of vaginal injections in normal labors. I always instruct my patients to take a warm bath as soon as the labor begins and to send for me immediately. Upon my arrival I prepare my solutions and thoroughly disinfect my hands and the genitalia of patient before making an examination. After satisfying myself that everything is all right, I refrain from further examinations until the labor is almost completed unless some complication arises. This I think a very essential precaution, for no matter how thoroughly the hand may be disinfected before each examination, there is always an element of danger which should be avoided. After delivery in normal cases, where it has been unnecessary for me to introduce my hand into the vagina or uterus, I use no injections, but simply wash off the parts thoroughly with bichloride solution 1:1000, and apply the "antiseptic pad," as recommended by Garrigues.

Where it has been necessary for me to use instruments or introduce my hand into the uterus, then I wash out the uterus thoroughly with about a quart of a 1:5000 bichloride solution, using a fountain syringe and a Chamberlain's glass tube. When any part of the membranes is retained it should be removed at once, either with the fingers or with, what is better in most cases, especially after abortion, the curette.

Another very important antiseptic measure is the immediate repair of the perineum when it has been rent. This is a very important precaution and should never, under any circumstances, be neglected when the condition of the patient will warrant any operative measures. I know physicians are frequently loath to tell their patients that they have been ruptured, on account of the unpleasant and generally undeserved criticisms to which it subjects them.

This, however, should never deter us from doing what is best for our patients, regardless of selfish interests. We should educate our patients in this direction and let them know that the accident is often unavoidable.

The surgical principle of thorough drainage is often neglected by obstetricians. My attention was first called to the subject by my friend, Dr. Virgil O. Hardon, of this city, and subsequent research and observation have convinced me of the correctness of his practice, which is, after normal labors, to instruct his patients to void their urine in the sitting posture, thus thoroughly emptying the uterus and vagina of all clots and *débris*.

Another advantage is that many patients are able to pass their urine who would otherwise require to have it drawn, which is always very unfortunate for the patient and disagreeable to the doctor.

In this paper I have not attempted an exhaustive discussion o the subject, but have briefly outlined those details which are applicable to private practice, and which I believe if faithfully, carried out, would save not a few lives and obviate many of the discomforts and dangers of the lying-in chamber.

To summarize:

1st. Disinfect thoroughly your hands and genitalia of patient before making a vaginal examination.

2d. Disinfect thoroughly all instruments which must come in contact with any part of parturient canal.

3d. Use vaginal injections before delivery where there is an unhealthy discharge or where the labor is protracted or instrumentation is necessary.

4th. If hands are introduced into vagina after delivery, use vaginal injections.

5th. If necessary to introduce hands or instruments into uterus before or after delivery, use intra-uterine injections.

6th. Apply "antiseptic pad" after thoroughly washing off the external parts with a 1:1000 bichloride solution.

7th. When necessary to introduce a catheter after delivery, always expose and thoroughly cleanse the parts beforehand.

8th. Where the antisepsis has not been thorough and puerperal fever supervenes, wash out the uterus not oftener than twice daily as long as indicated.

